# A Cupriavidus Pauculus Infection in a Patient with a Deep Brain Stimulation Implant

**DOI:** 10.7759/cureus.6104

**Published:** 2019-11-08

**Authors:** Mahesh B Shenai, Ramsey Falconer, Sean Rogers

**Affiliations:** 1 Neurosurgery, Inova Neuroscience Institute, Falls Church, USA; 2 Neurology, Inova Neuroscience Institute, Falls Church, USA

**Keywords:** deep brain stimulation (dbs), parkinson's disease, postoperative infection, cupriavidus pauculus

## Abstract

While deep brain stimulation (DBS) is now standard therapy for the treatment of Parkinson's disease, essential tremor, and dystonia, infections remain one of the most common perioperative complications. In this report, we describe a 58-year-old female with a history of medically refractory Parkinson's disease, who underwent magnetic resonance (MR)-guided bilateral subthalamic DBS. While the initial surgery and programming were successful, she returned in follow-up with signs of a generator pocket infection. She was taken to surgery for hardware explantation, and cultures revealed multispecies growth which included the rare Cupriavidus pauculus species. This is the first report of C. pauculus infection in conjunction with a neuromodulation device. We provide a literature review and discussion of C. pauculus, and its implications in the context of DBS surgery.

## Introduction

Deep brain stimulation (DBS) is a remarkable therapy to alleviate the cardinal symptoms of Parkinson’s disease. While largely successful, one of the most common risks associated with DBS includes infection, ranging from 2% - 10% of implantations, with gram-positive bacteria being the most frequent causative organism [1-3]. Suggested unique risk factors for infection include prior craniotomy, new indications [4] (e.g., Tourette’s, epilepsy), and even seasonality [3]. More recently, DBS intracranial lead implants have been surgically implanted while in a diagnostic magnetic resonance (MR) scanner, and infection risk in these cases are comparable to operating room placement [5].

The case below describes the unique case of a patient who developed a surgical site infection four months after battery implantation. Culture revealed a polymicrobial source, including the rarely identified Cupriavidus pauculus species. This is the first report of C. pauculus being associated with a DBS implantation or, more broadly, any surgical implant.

## Case presentation

The patient is a 64-year-old right-handed female with a history of Parkinson’s disease diagnosed 11 years prior, as well as an anxiety disorder. She initially presented with akinetic dystonia-predominant symptoms, affecting primarily her right side. Over the years, the dystonia worsened, with resulting cycling from severe dyskinesias to freezing episodes during the day to the point of disability. Though initially well-controlled on dopaminergic medications, she progressed to the point of becoming more and more resistant to control with oral medications, and “on-off” testing resulted in a reduction of the Unified Parkinson's Disease Rating Scale (UPDRS) Part III score from 78 to 32. Because of these factors, she was referred for bilateral subthalamic nucleus (STN) DBS.

**Figure 1:**
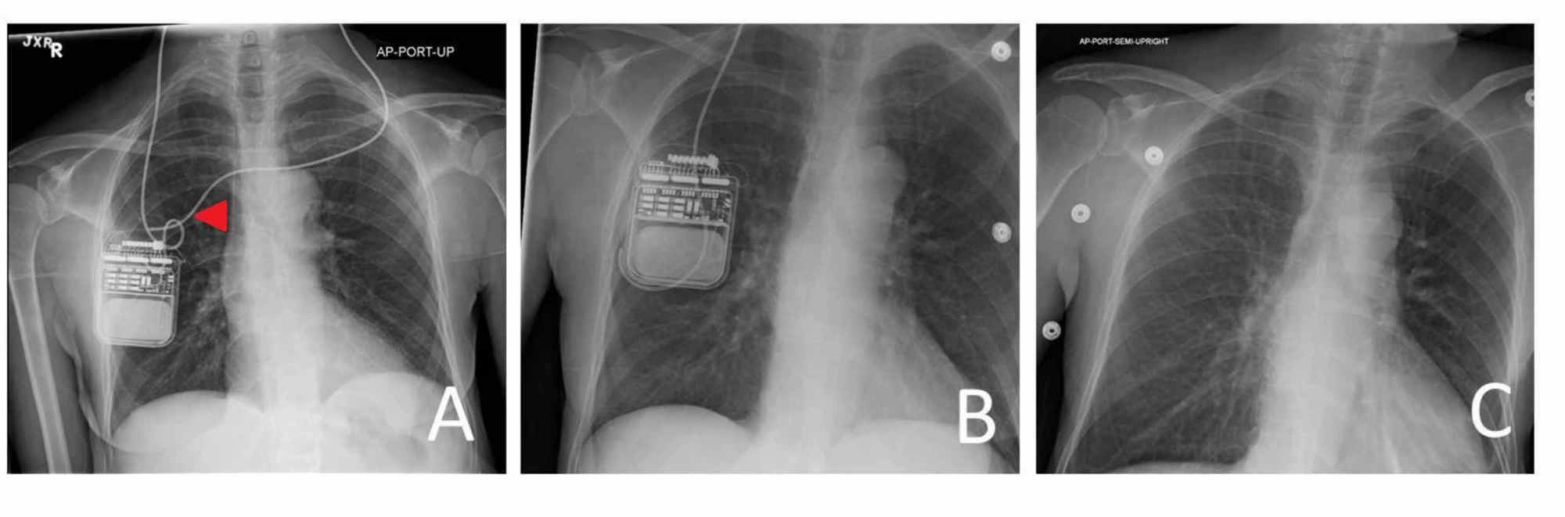
Management of DBS hardware infection (A) Original configuration of bilateral deep brain stimulation (DBS) connection to right subclavicular battery. Arrow indicates the affected lead, which is explanted; (B) Initial explantation of the left connector, maintaining the right-sided system; (C) Subsequent explantation of bilateral connecting leads and battery.

During initial surgical counseling, it was clear that she could not tolerate the “awake” frame-based surgery due to her anxiety and powerful dyskinesias. Therefore, MR-guided DBS was offered and she agreed. The patient underwent the procedure successfully in a diagnostic MR-scanner under general anesthesia and received prophylactic preoperative cefazolin, supplemented by local vancomycin powder. Medtronic 3389 intracranial leads were connected to a Medtronic Activa™ battery (Medtronic, Minneapolis, MN) in the operating room 19 days later (Figure 1A).

The patient returned for initial programming one month after intracranial implantation and demonstrated a notable and effective clinical response. Over three months, she also benefited from a reduction of dopaminergic medications and near full resolution of her dyskinesias. However, three months postoperatively, she presented to the ED with erythema and minimal serous drainage from the left scalp staging incision. She was afebrile at this time. She was noted to have multiple insect bites across her lower extremities and chest that she identified as “chigger” bites from camping in the woods. The incision was locally cleansed, and she was placed on a two-week course of Augmentin. 

She returned to the clinic a month later, with erythema and a fluctuant mass over the DBS generator in the right subclavicular region. She admitted to swimming in a “homemade hot tub” in the woods, filled with lake water and sterilized by bleach. Given the physical appearance of the surgical site and the interval history, she was taken to the operating room for explantation. The left connecting lead was severed distal to the connector at the staging incision, and the right subclavicular incision was opened and exposed, resulting in the egress of non-purulent serous fluid. The left lead connector was removed (Figure 1B). The wound site and serous fluid were cultured for aerobic and anaerobic organisms. Given her good response to the DBS device and the non-purulent fluid collection, the battery (connected only to the right intracranial lead) was reimplanted, sprinkled with vancomycin powder, and closed.

She did well postoperatively, and an infectious disease consultation was obtained. Wound cultures revealed a light-growth polymicrobial infection, including the species methicillin-sensitive Staphylococcus aureus (MSSA), Enterobacter amnigenus, and Cupriavidus pauculus. The lab described the C. pauculus growth of “questionable significance due to low quantity.” However, the infectious disease consultant believed this was a clinically significant species in this case, and she was treated with a six-week course of intravenous ceftazidime. Nevertheless, it was decided to keep the battery in place, as the cultures were not convincing. She was discharged.

She returned to the emergency department two months later with wound dehiscence and purulence over her right-sided generator. Again, she admitted to non-compliant behaviors, including camping in the woods, with minimal attention to basic wound care. Subsequently, the generator and the connecting lead were explanted (Figure 1C). The operative wound cultures remained negative. She was placed empirically on cefepime for two weeks. She has since declined the reconnection of the intracranial leads to a generator.

## Discussion

Infections are one of the most common complications in DBS surgery and can range from superficial skin manifestations to deeper and fulminant intracranial infections. Most DBS-related infections occur in the first three months after surgery, though the presentation is possible in later periods as well. Treatment ranges from an empiric course of antibiotics to full explantation of the entire DBS system [5].

This case initially presented as a typical surgical site infection, with attention paid to the left cranial staging incision overlying the connector. There were no clear signs of infection at this time; however, it became apparent that the patient was participating in several outdoor activities, including sleeping on the dirt ground and utilizing a “homemade” hot tub, described as lake water cleaned with bleach. At this point, the serous drainage derived from her extensive “chigger” bites was thought to result from trombiculid mites. However, later presentation of the generator pocket fluctuance clearly indicated an infection process. This patient’s history of immersion in the “homemade” pool raised significant suspicion of the contamination of her relatively fresh surgical incision with this species, despite the laboratory’s questioning of its significance.

Wound cultures from the operating room revealed C. pauculus, a rare gram-negative bacillus, that has been described as a “water” species responsible for rare nosocomial infections and environmental contamination. Case reports of both community-acquired [6-7] and ventilator-associated pneumonia [8], meningitis, and septicemia [9] have been described, usually in immunocompromised patients. Association with extracorporeal membrane oxygenation (ECMO), which contains a water reservoir, have also been reported as a source of bacteremia [10]. Resistance to meropenem and other antibiotics have been described [11].

Environmentally, C. pauculus has been found in soil and water collections. In 2010, a minor outbreak was reported in Ohio, in which a clinical microbiology lab identified 27 cases of C. pauculus over six weeks, from a single physician’s office that moistened culture swabs with tap water [12]. Interestingly, C. pauculus was also identified in the International Space Station potable water supplies during pre-consumption testing [13]. Based on these sources and case reports, it is conceivable that C. pauculus contaminated the surgical wound and resulted in a clinically-relevant infection. 

C. pauculus has never been described in association with DBS nor any similar surgical implant, though it is certainly possible that the other present species (MSSA, Enterobacter) could have been the causative agent of infection, though it is impossible to prove. This patient was not immunocompromised, but the rare presence of C. pauculus in the polymicrobial roster, at the very least, suggests the mechanism of infection resulting from non-compliance with postoperative instructions. This instance highlights the need to emphasize postoperative wound care instructions and activity restrictions, which can be occasionally taken for granted. 

## Conclusions

This case report describes the rare finding of C. pauculus in association with a recent DBS implantation, likely inflicted through contaminated water penetration through the surgical wound. We believe this is the first report of the C. pauculus species being associated with any type of permanent surgical implant. While C. pauculus itself is rare, this case highlights the importance of strict wound care and activity restrictions postoperatively, particularly in the case of DBS where infections have a high financial and clinical cost.
